# Environmental Sustainability Assessment of a Filtration–Diafiltration Strategy for Recovering Savory Compounds from Mussel Cooking Water

**DOI:** 10.3390/membranes15080242

**Published:** 2025-08-08

**Authors:** Erasmo Cadena, Jo Dewulf, David San Martin, Jone Ibarruri, Bruno Iñarra, Monica Gutierrez

**Affiliations:** 1 Sustainable Systems Engineering (STEN), Department of Green Chemistry and Technology, Faculty of Bioscience Engineering, Ghent University, Coupure Links 653, B, 9000 Ghent, Belgium; jo.dewulf@ugent.be; 2 AZTI, Food Research, Basque Research and Technology Alliance (BRTA), Parque Tecnológico de Bizkaia, Astondo Bidea, Edificio 609, 48160 Derio-Bizkaia, Spain; dsanmartin@azti.es (D.S.M.); jibarruri@azti.es (J.I.); binarra@azti.es (B.I.); mgutierrez@azti.es (M.G.)

**Keywords:** savory compounds recovery, nanofiltration system scale-up, volumetric concentration factor, environmental sustainability, Life Cycle Assessment, seafood side streams, carbon footprint, freshwater eutrophication

## Abstract

Global seafood production and consumption have increased in recent years, leading to a significant rise in side streams. Process waters are often disposed as wastewater, causing difficulties for industries in meeting the discharge standards. This is particularly relevant to the mussel processing industry, where one-third of the raw material ends up as high-organic content effluent. This study aims to optimize a nanofiltration–diafiltration (NF–DF) strategy to recover valuable savory compounds from mussel cooking water, to reduce the effluent organic pollution, and to evaluate its environmental sustainability using Life Cycle Assessment. Pilot trials lead to a configuration, combining a volumetric concentration factor of 10 in NF and 20 in DF, which achieved enhanced protein concentration (1.5-fold), amino acid concentration (5.2-fold), and COD removal (98.2%). The environmental assessment highlighted electricity consumption during NF and DF as the primary environmental hotspot, resulting in a carbon footprint of 0.12 kg CO_2_ eq. kg^−1^ of savory compounds and water use of 0.65 m^3^ deprived kg^−1^. Prospective scenarios projected that ongoing energy system transitions could significantly reduce climate change and acidification impacts by over 75% by 2050. The proposed NF–DF strategy enhances resource efficiency and sustainability in seafood processing by recovering high-value compounds and facilitating water reuse.

## 1. Introduction

Global seafood consumption has seen marked increase in recent years, resulting in an average consumption of 20 kg per capita annually, driven by growing awareness of its health benefits and the demand for more sustainable and ethical sources of protein [1]. Among seafood products, mussels have garnered particular attention due to their rich nutritional profile, including high-quality protein, Omega-3 fatty acids, vitamins, and essential minerals, as well as their reputation as one of the most environmentally sustainable forms of aquaculture. Reflecting this trend, organic aquaculture production in the EU rose by 60% between 2015 and 2020, with mussels being a major contributor to this growth [2]. However, despite these benefits, there are also challenges associated with increasing mussel consumption. These include ensuring the safety and quality of the product, managing the environmental impacts of mussel farming, and processing while meeting the growing demand without compromising the oceans’ health [3].

Mussel aquaculture now accounts for over 90% of global mussel supply and is widely recognized as a low-impact form of food production. However, the processing of mussels still presents significant environmental and efficiency challenges. Industrial mussel processing is highly resource-intensive and yields substantial waste, with only about 30% of the biomass reaching the consumer as edible product [4].

While the valorization of solid side streams, such as shells and meat residues, has advanced considerably in recent years, resulting in applications in animal feed, cosmetics, and nutraceuticals [4], the potential of liquid side streams remains underexplored. One particularly promising liquid side stream is mussel cooking water (MCW), a nutrient-rich effluent generated during thermal processing. Unlike early-stage wastewater streams (e.g., from washing or debyssing), cooking water is produced under hygienic conditions and contains high concentrations of peptides, amino acids, and flavor compounds [5,6]. This makes it a viable candidate for valorization into food-grade ingredients, offering both economic potential and opportunities for improved environmental performance through waste minimization [4].

Among the available technologies for recovering valuable compounds from such liquid streams, membrane-based concentration, particularly nanofiltration, has emerged as a promising solution due to its relatively low energy use and ability to retain medium- to high-molecular weight solutes [7]. While methods such as electrodialysis and reverse osmosis have also been investigated for desalination and concentration [8,9,10], they often require intensive pretreatment and may suffer from fouling and efficiency losses in the presence of organic matter.

Nanofiltration (NF) has emerged as a versatile membrane technology for the recovery of valuable compounds, such as polyphenols, proteins, peptides, and organic acids, from food industry wastewaters and process streams. Owing to its selective molecular separation capability, NF efficiently concentrates bioactive molecules and nutrients, supports water reuse, and reduces pollutant loads. These features make it well suited for promoting circular economy practices in agri-food processing [11,12]. Recent research reports high retention rates of target compounds and confirms the suitability of NF for valorizing a wide range of residual feedstocks, underscoring its potential for sustainable resource management in the food sector [11,12,13]. Specifically, in the context of seafood processing, NF exhibits favorable selectivity for protein fractions while limiting salt retention. However, partial loss of low molecular weight compounds (<400 Da) can occur during filtration [10,14,15]. The use of NF membranes with a 150–300 Da of Molecular Weight Cut-Off (MWCO) can retain up to 92% of free amino acids present in fish protein hydrolysates [16].

The use of liquid by-products, specifically from MCW side streams, is considered a potential solution to enhance circularity in the seafood industry. However, it is essential to assess the environmental implications of such valorization strategies. The food sector, particularly animal protein production, is a major contributor to environmental issues such as climate change, water consumption, biodiversity decline, and habitat loss [17,18]. To address these concerns, a thorough analysis of production processes is vital. In this regard, Life Cycle Assessment (LCA), a technique used to quantitatively gauge environmental impacts, stands out as a valuable analytical tool. LCA helps to identify areas for environmental improvement and provides critical information to the stakeholder [19]. Despite its relevance, few LCA studies have addressed valorization strategies for liquid effluents in the seafood industry, particularly for mussel processing [20,21,22,23,24].

Extending the findings of previous research that demonstrated the technical feasibility of using nanofiltration to recover savor-enhancing compounds from mussel cooking water at a semi-pilot scale [25], the present study advances this research by scaling up the process using representative volumes and refining operational parameters for practical industrial implementation. In addition to technical optimization, this work is the first to apply Life Cycle Assessment (LCA) to evaluate the environmental sustainability of MCW valorization. By combining experimental process development with LCA, this study provides a comprehensive assessment of both technical and environmental performance. As such, it offers novel insights into the potential of MCW valorization to support circular economy strategies in seafood processing, an area that remains largely underexplored compared with the valorization of solid side streams [22].

## 2. Materials and Methods

### 2.1. System Description

Validation tests were performed at the pilot scale in the facilities of Pescados Marcelino (Galicia, Spain) as part of the EU project WaSeaBi [24]. Pescados Marcelino is a seafood processing company that produces ready-to-eat mussels (*Mytilus galloprovincialis*) through steam cooking. Three individual batches of mussel cooking water (MCW), each measuring 1 m^3^, were hygienically collected directly from the cooking system outlet at 100 °C and promptly cooled to 40 °C. To avoid unnecessary energy consumption, the stream was only cooled down to 40–50 °C, which is a suitable temperature for membrane filtration systems. This approach helps preserve the aromatic compounds present in the cooking water and reduces the overall processing time. Prior to processing, initial microbiological and compositional analyses were performed to verify sample quality.

### 2.2. Concentration Process

**Figure 1:**
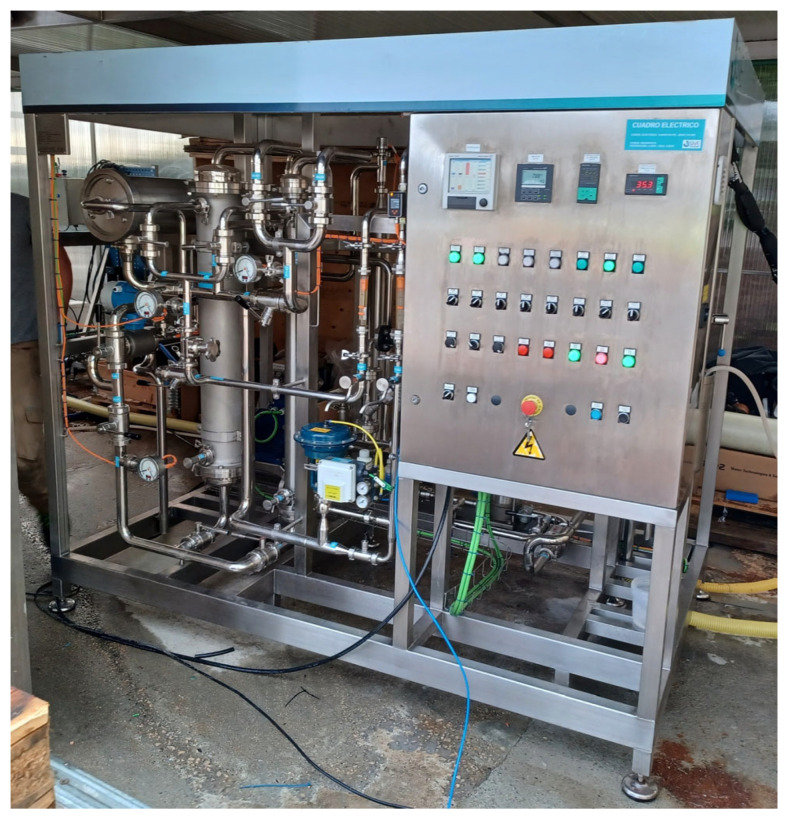
NF pilot plant working in the Pescados Marcelino factory during the assays.

The concentration of MCW was performed using a nanofiltration membrane system designed to retain organic molecules while allowing salts and water to pass through. The process consisted of two main steps: nanofiltration (NF) and diafiltration (DF) with the objective of obtaining concentrates rich in savory compounds such as glutamic acid, while simultaneously reducing salt content (Figure 1).

The process was also carried out at the Pescados Marcelino facility using a multipurpose membrane filtration pilot plant (SIVE, Spain) equipped with a nanofiltration membrane with a Molecular Weight Cut-Off of 150–300 Da (Parker, Cleveland, OH, USA. Ref. NF ATF8038) and a 200 µm prefilter (Figure 1).

**Figure 2:**
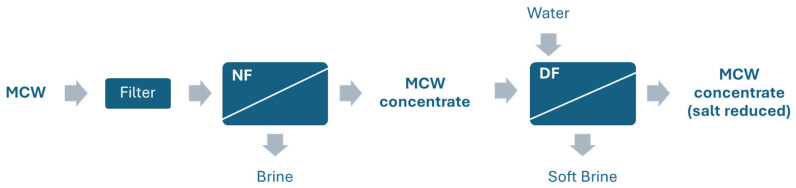
Schematic representation of the mussel cooking water (MCW) recovery process comprising nanofiltration (NF) and diafiltration (DF). MCW is prefiltered to remove solids, and then processed by NF to obtain clean brine and NF concentrate that undergoes DF with added water, yielding soft brine and a final product enriched in salt compounds. Arrows indicate material flow direction.

In the first step, NF was applied to concentrate proteins while maintaining the initial salt concentration. The DF step followed, in which the NF concentrate was diluted with water to recover the original protein concentration, after which a second nanofiltration was performed to further reduce the salt content in the final concentrate (Figure 2). Filtrations were conducted under the following operating conditions for both NF and DF: tangential flow mode, temperature at 40–50 °C, and operating pressure ranging from 12 to 20 bar [25].

The volumetric concentration factor (VCF), defined as the ratio between the initial volume and the final volume of the concentrate, is a key parameter for determining the optimal concentration level without inducing excessive membrane fouling, which would reduce permeate flux [26]. In a previous work [25], the membrane filtration system was operated in batch mode. Therefore, one of the main objectives of the present work was to optimize the VCF under continuous filtration conditions, aiming to maximize the yield of both protein-rich concentrate and regenerated water, while minimizing energy consumption, membrane fouling, and cleaning frequency. Membrane cleaning operations follow a conventional scheme involving an alkaline cleaning step using a NaOH-based detergent, followed by an acid cleaning step with a citric acid-based detergent, aimed at restoring membrane performance.

To evaluate and optimize the nanofiltration–diafiltration strategy, three experimental assays were designed (Table 1), each testing different VCF configurations. Due to the limited availability of MCW and the constraints of an industrial setting, each experiment was performed only once. These single-run assays were designed to assess the influence of varying concentration levels on system performance and product quality. The selected configurations aimed to explore trade-offs between concentration efficiency, compound recovery, and energy demand, providing a foundation for identifying the most suitable operational strategy for future industrial scale-ups.

### 2.3. Analytical Methods

The proximate composition of the samples was analyzed to validate and verify the technologies assessed for the recovery of savory compounds and water regeneration, according to the Association of Official Analytical Chemists [27]. The dry matter of the samples was determined by drying them at 100 °C to a constant weight (method 934.01). Crude protein content was determined by Kjeldahl methodology using a conversion factor for nitrogen to protein of Nx6.25 (method 955.04). Chemical Oxygen Demand (COD) was analyzed following standardized methods [28].

The free amino acid (Aa) profile of the samples was determined using high-performance liquid chromatography with fluorescence detection (HPLC/FD). Sample preparation involved derivatization with the AccQ-Fluor Reagent Kit (Waters, Milford, MA, USA) following neutralization with 6 N HCl for 24 h at 100 °C. Chromatographic separation was performed using an Agilent Polaris C18 column (4 μm, 83.9 × 150 mm) (Agilent Technologies, Madrid, Spain). The analysis was carried out under a gradient elution system: 60% acetonitrile and 40% AccQ-Tag buffer (10% *v*/*v*) for the initial 5 min, followed by 100% Waters AccQ-Tag buffer (10% *v*/*v*) for the subsequent 9 min. Fluorescence detection was set at 250 nm for excitation and 396 nm for emission. Amino acid identification was achieved by comparing sample chromatograms with standard curves prepared using commercial amino acid standards (AccQ-Tag, Pico-Tag, Accu-Tag Ultra; Waters, Milford, CT, USA). The amino acid profile was expressed both as a percentage (grams of amino acid per 100 g of protein) and in concentration in the sample (mg L^−1^) [29]. All physicochemical analyses were performed in triplicate.

### 2.4. Data Interpretation

Statistical analysis of experimental factors was conducted using ANOVA (analysis of variance), with significance established at a *p*-value threshold of <0.05. The assumption of normality was assessed using the Shapiro–Wilk test, while homogeneity of variances was evaluated with Levene’s test. In cases where data did not meet the assumptions of normal distribution or equal variances, the non-parametric Kruskal–Wallis test was applied for a group of comparisons. Post hoc multiple comparisons were performed using Tukey’s Honestly Significant Difference (HSD) test.

All statistical analyses were carried out using Statgraphics Centurion XVI version 16.2.04 (Statgraphics Technologies, Inc., the Plains, VA, USA).

### 2.5. Life Cycle Assessment

As introduced earlier, the second objective of this study is to evaluate the environmental impacts associated with the proposed strategy for recovering savory-rich compounds from MCW using nanofiltration-based membrane technology from a life cycle perspective. The assessment follows the international standards ISO 14040:2006 [19] and ISO 14044:2006 [30], which provide a systematic framework comprising goal and scope definition, life cycle inventory analysis, impact assessment, and interpretation.

#### 2.5.1. Goal and Scope

The LCA aims to quantify the environmental performance of a pilot-scale membrane filtration process designed to valorize MCW into a glutamate-rich concentrate. The objectives were to (i) identify environmental hotspots across the process chain and (ii) evaluate potential future environmental impacts under evolving energy and material scenarios.

**Figure 3:**
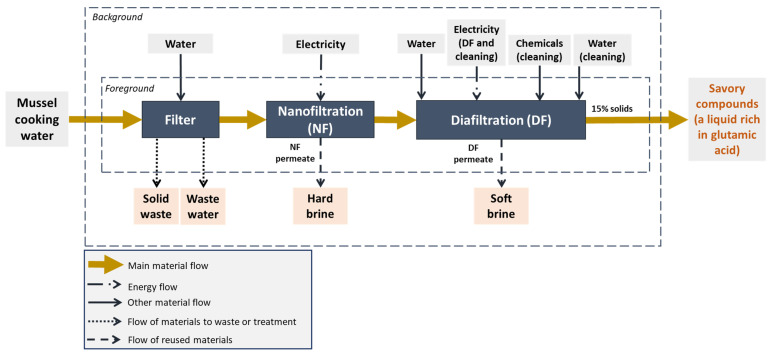
System boundaries definition of the membrane concentration technology. NF: nanofiltration; DF: diafiltration.

The functional unit (FU) is defined as 1 kg of savory compound concentrate (15% solids, rich in glutamic acid), produced from mussel cooking water. The system boundaries are set from cradle to gate (Figure 3), encompassing all inputs and operations up to the point where the concentrate is ready for packaging. Recovered water from diafiltration and nanofiltration steps is internally reused, reducing the freshwater demand. Membranes are reused for internal non-critical cleaning processes after a 4-year operational lifespan, and thus, their end-of-life impacts are considered negligible within this assessment.

#### 2.5.2. Life Cycle Inventory

Primary inventory data were collected from the pilot facility, which processes 4,840,000 L of mussel cooking water per year and yields approximately 240,571 L of concentrated product. Background data were sourced from ecoinvent v3.10, applying the cut-off system model, which reflects market-average and current technology conditions. Datasets representing the European market (RER, market for) were utilized for all processes, with three exceptions: sodium hydroxide (NaOH) and citric acid, for which only global (GLO) data were available, and electricity, for which the Spanish electricity mix at medium voltage was applied.

To assess potential future performance, the premise v2.2.5 tool was used to generate prospective versions of the ecoinvent database for 2030, 2040, and 2050, based on the SSP2-NDC scenario from the REMIND v2.1 Integrated Assessment Model (IAM) [31]. The premise dynamically updates background inventories (electricity, transport, steel, and cement) to reflect anticipated improvements in emissions and efficiency. Only REMIND was used in this study to avoid redundancy, as short-term differences between IAMs remain limited.

Table 2 presents the life cycle inventory associated with the defined functional unit.

#### 2.5.3. Life Cycle Impact Assessment

The assessment was conducted using Activity Browser v2.11.2, a graphical interface built for the Brightway2 LCA framework, which supports complex foreground–background model integration and uncertainty analysis [33]. The impact assessment method used was Environmental Footprint (EF) v3.1, recommended by the European Commission. The analysis focused on four key midpoint indicators relevant to seafood processing and membrane-based valorization systems: climate change (GWP 100 years), acidification, freshwater eutrophication, and water use. These impact categories were selected due to their direct relevance to the assessed system: greenhouse gas emissions associated with electricity generation, the potential release of acidic substances during chemical cleaning, nutrient loads in wastewater discharges, and significant water consumption across filtration and cleaning processes. Together, they provide a representative picture of the most relevant environmental pressures linked to membrane filtration technologies in the seafood industry [34,35,36].

#### 2.5.4. Life Cycle Impact Interpretation

A contribution analysis was performed for all assessments to identify the main contributors to the selected impact categories. To account for uncertainty in background data, a Monte Carlo simulation with 1000 iterations was performed in Activity Browser. The simulation applied probabilistic distributions to ecoinvent datasets (e.g., electricity production, chemicals), while keeping pilot-level foreground data deterministic.

A prospective sensitivity analysis was conducted to explore how the environmental performance of the valorization process may evolve over time. This analysis was based on projections for the years 2030, 2040, and 2050 using the Integrated Assessment Model (IAM) REMIND v2.1 (Regional Model of Investments and Development) [37]. REMIND integrates socio-economic, technological, and policy dimensions to simulate long-term interactions between the energy system, economy, land use, and climate change. The selected scenario, SSP2-NDC, reflects a middle-of-the-road development pathway aligned with current nationally determined contributions. It encompasses not only progressive decarbonization of the electricity mix, but also advances in energy efficiency, technological deployment, and broader systemic transformations [37]. By incorporating these changes, the prospective analysis enables a forward-looking evaluation of the potential environmental benefits of the MCW valorization technology under more sustainable future conditions.

## 3. Results and Discussion

### 3.1. Concentration Processes

To evaluate the performance and scalability of the proposed system for recovering protein content from MCW and simultaneously obtaining regenerated saline water with reduced COD levels, three trials were conducted with varying VCFs (see Table 1). In Assay 1, both nanofiltration (NF) and diafiltration (DF) were operated at a VCF of 10. Assay 2 maintained the same VCF for NF (10×) but increased the VCF of DF to 20×, aiming to enhance the protein concentration. In Assay 3, the NF step was intensified to a VCF of 20×, followed by a planned DF step at the same VCF. While the NF stage in Assay 3 was completed, the system experienced significant fouling, resulting in a very low process yield, which prevented the DF step from being carried out.

As mentioned above, the assays were carried out using representative volumes of MCW (1 m^3^), and the results were analyzed in terms of concentration of target compounds in the retentate, permeate quality, membrane performance, and operational stability. The findings provided valuable insight into the optimization of the concentration process.

**Figure 4:**
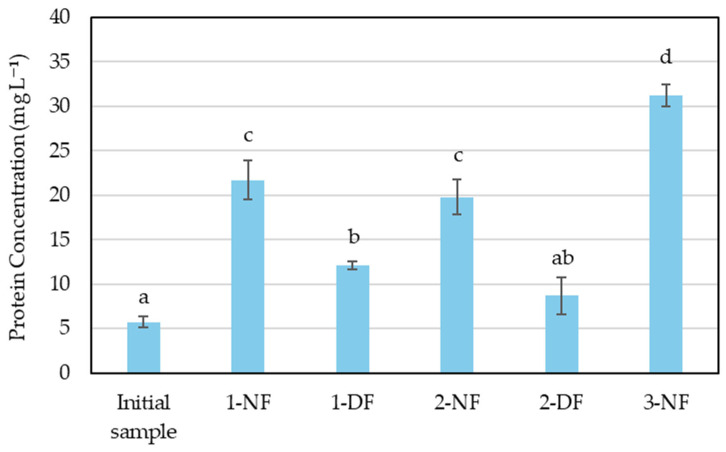
Protein content in MCW concentrates through different pilot assays. Initial sample of MCW and the resulting concentrates: Assay 1: 1-NF (nanofiltration 10×) and 1-DF (diafiltration 10×); Assay 2: 2-NF (nanofiltration 10×) and 2-DF (diafiltration 20×); and Assay 3: 3-NF (nanofiltration 20×). SD (*n* = 3). Same letter means no significant difference between samples at 95% confidence.

Figure 4 shows the results obtained for protein concentration in the retentates across the different trials to know the potential of nutritional properties and the capacity as a savory compound.

The protein concentration results across the different scaling trials reveal a clear trend: the highest concentrations are consistently achieved after the NF step. In Assay 1, the protein level increased from an initial 5.73 mg L^−1^ to 21.70 mg L^−1^ after NF, before decreasing to 12.15 mg L^−1^ following the DF step. A similar pattern was observed in Assay 2, with NF yielding 19.80 mg L^−1^ and DF reducing it to 8.70 mg L^−1^. Assay 3, which only completed the NF step, recorded the highest protein concentration of all trials at 31.25 mg L^−1^. These results confirm that NF is the most effective stage for protein concentration. However, due to the pore size range of the NF membranes (150–300 kDa), a portion of the smaller protein fractions, such as low molecular weight peptides and free amino acids, pass into permeate, leading to partial protein loss.

This observation is consistent with previous research related to the food industry, where nanofiltration membranes have been shown to allow the passage of low molecular weight compounds such as amino acids and small peptides due to their relatively large pore size, resulting in partial protein loss during the filtration process [38].

**Figure 5:**
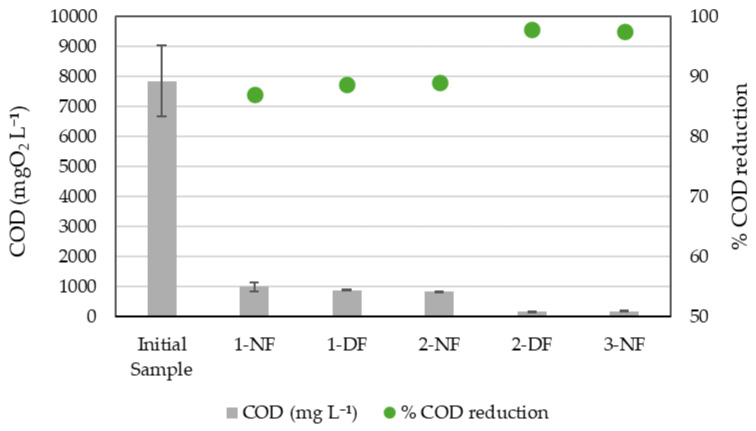
COD concentration in the different permeate steps during the assays. It includes the initial COD in MCW and the resulting permeates after Assay 1: 1-NF (nanofiltration 10×) and 1-DF (diafiltration 10×); Assay 2: 2-NF (nanofiltration 10×) and 2-DF (diafiltration 20×); and Assay 3: 3-NF (nanofiltration 20×). Error bars represent SD (*n* = 3).

The Chemical Oxygen Demand (COD) values of the resulting regenerated effluents demonstrate a clear reduction in organic load throughout the filtration process (Figure 5). In all assays, the NF step led to a significant decrease in COD levels, with further reductions observed after the DF step in Assays 1 and 2. The COD concentration dropped from the initial sample value to progressively lower levels in the NF and DF permeates, indicating effective removal of organic matter.

When a VCF of 10× was applied in the NF step (1-NF and 2-NF), the removal of COD was from 85 to 91% and the final COD concentration was around 850 mgO_2_ L^−1^. However, when a factor of 20× was applied in the NF step (3-NF), COD reduction increased up to 97.4% (final COD of 195 mgO_2_ L^−1^). The application of a DF 10× to the NF 10× sample only achieved a further slight COD reduction of 2% (1-DF, 87%). Conversely, the application of a VCF of 20× in the DF step after the NF 10× produced a significant COD removal increase up to 98.2% and a final COD in effluents of 166 mgO_2_ L^−1^.

The increase in total free amino acids has been reported previously [25], and it is attributed to shear forces during membrane filtration, which break down proteins and peptides and generate new free amino acids. In the NF step, the shear force raises free amino acids levels, while DF causes some of these amino acids to pass into permeate, increasing the organic matter in the DF permeate. Furthermore, salt reduction in the second step contributes to improve filtration performance. This fact explains higher VCF values and therefore a high level of concentration [39].

These results align with a previous work [25] and other published studies demonstrating that nanofiltration, particularly when combined with diafiltration, is highly effective in reducing organic load in food industry wastewater, achieving substantial COD removal while operating under relatively low pressure conditions [38,40].

The final permeate generated during the nanofiltration and diafiltration process is a salty sterilized liquid that allows its reuse for cleaning purposes.

To complement the evaluation of protein recovery and organic matter removal from effluents, energy consumption was also assessed across the three experimental trials. This analysis provides critical insight into the operational efficiency and sustainability of the proposed system under different concentration regimes.

By comparing the specific energy demand associated with each configuration, it is possible to identify the most energy-efficient strategy for maximizing protein concentration while minimizing environmental impact.

Energy consumption per cubic meter of MCW treated varied notably across the three experimental trials, reflecting the impact of different concentration strategies on operational efficiency (Table 3).

In Assays 1 and 2, where both the NF and DF steps were completed, the total energy demands were 16.42 and 17.12 kWh m^−3^, respectively. These values indicate a relatively balanced energy profile between the NF and DF stages. In contrast, Assay 3, in which only the first step was completed due to excessive fouling, exhibited the highest energy consumption at 21.51 kWh m^−3^. This sharp increase is attributed to the intensified NF operation (VCF = 20) highlighting the trade-off between higher concentration factors and energy efficiency. These findings underscore the importance of optimizing process parameters not only for performance but also for energy sustainability.

Effluent generation was also evaluated to assess the environmental impact of each process configuration (Table 4). When normalized per cubic meter of treated MCW, a slight increase in the volumes of the final effluent is calculated—1800, 1850, and 1900 L m^−3^, respectively. This trend is even more pronounced when scaled to daily production (20 m^3^ of MCW per production day), with Assays 1, 2, and 3 generating 36, 37, and 38 m^3^ of effluent, respectively. However, it is important to note that, in Assay 3, the DF step was not carried out due to excessive membrane fouling and the loss of permeability during the NF stage, which rendered further processing unfeasible. As a result, the reduced effluent volume in this case reflects an incomplete process rather than an optimized configuration. These results highlight the potential of higher VCF configurations to substantially reduce effluent discharge, contributing to improved water management, and the need to take into account the fouling processes and hence the importance of performing pilot studies to correctly evaluate the real environmental impact of processes.

### 3.2. Effect of Membrane Processing on Glutamic Acid Recovery

The recovery of savory compounds from MCW was evaluated based on the glutamic acid contained in the concentrates from the different experimental assays. Despite operating at a higher concentration yield during the NF step in Assay 3 (VCF = 20), the resulting protein content in the concentrate was not significantly higher than in the previous trials. Moreover, the process suffered from severe membrane fouling, which not only decreased the permeate flow but also introduced high variability in the results, as reflected by the high standard deviation. Consequently, this configuration was deemed inefficient and was not considered for glutamic acid quantification.

The following section focuses on the outcomes from Assays 1 and 2, where both NF and DF were successfully completed. Table 5 presents the distribution of free amino acids, expressed as concentration, across the initial MCW sample and the concentrates obtained after the NF and DF steps in Assays 1 and 2.

In both assays, NF led to a marked increase in the concentration of major amino acids, such as aspartic and glutamic acids, with Assay 2 consistently showing higher values due to the more intensive concentration process. For instance, glutamic acid increased from 81.8 mg L^−1^ in the initial sample to 567.4 mg L^−1^ in Assay 1 and 673.7 mg L^−1^ in Assay 2 after NF. However, DF reduced these concentrations, although Assay 2 still retained higher levels (441.4 mg L^−1^ vs. 366.0 mg L^−1^ in Assay 1). Similar trends were observed for aspartic acid. In contrast, amino acids like asparagine and serine showed moderate increases after NF but experienced sharp reductions after DF, particularly in Assay 2, suggesting a greater sensitivity to diafiltration at higher concentration levels.

Further differences between the two assays were observed in specific amino acids. Glutamine, for example, increased significantly after NF in Assay 2 (190.9 mg L^−1^) but dropped drastically to 2.2 mg L^−1^ after DF, indicating its high susceptibility to removal during diafiltration. Proline also showed a sharp increase after NF in Assay 2 (181.7 mg L^−1^), and although its concentration decreased after DF, it remained higher than in Assay 1 (98.3 mg L^−1^ vs. lower values). Overall, NF consistently enhanced amino acid concentrations due to the concentration effect, with Assay 2 showing greater increases. However, DF introduced a selective effect, reducing the concentrations of certain amino acids more significantly, especially in the more concentrated samples. These results highlight the importance of balancing concentration intensity with retention efficiency when targeting specific amino acids for recovery.

Table 6 reports the amino acid distribution as percentages, a representation that facilitates a more accurate assessment of a membrane’s differential selectivity toward the various amino acids. A general trend observed in both assays is the increased relative abundance of aspartic and glutamic acids following the concentration steps, particularly Assay 2. For instance, glutamic acid rose from 7.2% in the initial sample to 21.9% after DF in Assay 1 and further to 27.7% in Assay 2. Conversely, amino acids such as asparagine and serine showed a marked decrease in their relative proportions, with asparagine dropping to just 1.0% after DF in Assay 2.

When comparing the two assays, Assay 2 exhibited higher percentages of dominant amino acids, like glutamic and aspartic acids, reflecting the effect of the more intensive 20× concentration process. In contrast, minor amino acids, such as asparagine and serine, were further reduced in relative abundance, suggesting that higher concentration factors may disproportionately affect their retention. The DF step also played a selective role in reducing the relative content of certain amino acids like glutamine and serine, while enhancing the dominance of others such as glutamic acid. These results indicate that both the concentration factor and the DF process influence the amino acid profile of the recovered concentrate, with implications for the functional and sensory properties of the final ingredient. Similar results were obtained in a preliminary study carried out using batch nanofiltration equipment for the recovery of valuable compounds from MCW [25]. Some authors reported that the distribution of amino acids between retentate and permeate is determined by molecular size and specific interactions with a membrane, making NF membranes optimal for the selective concentration of amino acids in fish processing streams [16,41]. Glutamic acid is one of the major amino acids present in fish protein streams, and its concentration increases in the NF retentate after fractionation. Saidi and Ben Amar [42] presented a study on tuna processing waste that reported effective retention of glutamic acid—along with aspartic acid, glycine, alanine, valine, and leucine—in NF retentate.

### 3.3. Optimal Concentration Strategy of Proposed Nanofiltration System

Among the three experimental configurations tested, Assay 2 emerged as the most balanced and effective strategy for optimizing the nanofiltration-based concentration process. This configuration, which combined an NF step at VCF 10× with a more intensive DF step at VCF 20×, achieved notable improvements in protein and amino acid recovery, particularly for key compound glutamic acid. Compared with Assay 1, Assay 2 retained higher concentrations of selected amino acids even after the DF step, indicating enhanced selectivity and retention under intensified conditions. Furthermore, Assay 2 demonstrated the highest COD removal efficiency (97.9%), significantly reducing the organic load in the final effluent and supporting its potential for water reuse applications. These results highlight the configuration’s ability to maximize both product quality and environmental performance.

In addition to its compositional advantages, Assay 2 also maintained operational feasibility and energy efficiency, with a total energy demand of 17.12 kWh m^−3^, only slightly higher than Assay 1, and substantially lower that the 21.51 kWh m^−3^ recorded in Assay 3, which suffered from severe membrane fouling. Unlike Assay 3, Assay 2 successfully completed both the NF and DF steps without compromising membrane integrity or process stability. Moreover, the effluent volume generated was comparable between assays, resulting in no additional burden on downstream water management. Taken together, these findings position Assay 2 as the most promising candidate for industrial scale-up, offering a robust compromise between concentration performance, energy consumption, and effluent quality.

### 3.4. Life Cycle Assessment Results

**Figure 6:**
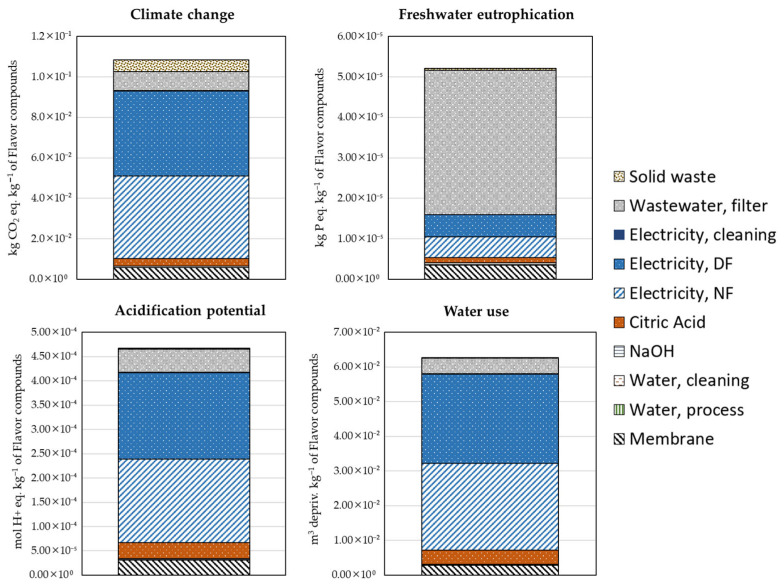
Environmental impact results for the production of 1 kg of savory compounds (functional unit) via a membrane concentration technology.

This section presents and discusses the LCA results for the four selected environmental impact categories. The results are based on data from Assay 2, which was selected for the assessment due to its best technical performance among the tested configurations. Figure 6 shows the contribution of various process inputs and outputs to the overall environmental impacts of the analyzed recovery strategy.

The membrane filtration process exhibited a carbon footprint of approximately 0.107 kg CO_2_ eq. kg^−1^ of savory concentrate produced. Electricity use in the NF and DF processes was the predominant contributor, accounting for roughly 82% of the total climate change impact. This high contribution is associated with the significant electricity requirements to maintain operational pressure and temperature conditions during filtration. These processes are energy-intensive, and the reliance on the Spanish electricity grid, which still includes a considerable proportion of fossil fuels, magnifies their environmental impact [43]. As of 2021, natural gas holds a significant position within the electricity mix, constituting 25% of the overall composition [44].

Chemical consumption, specifically citric acid used for cleaning, represented the second-largest contributor (around 8%) due to emissions associated with chemical manufacturing processes [45,46,47]. Contributions from wastewater treatment, membrane manufacturing, and solid waste management were minimal (<10%), highlighting electricity and cleaning chemical inputs as priority hotspots for potential improvements.

The acidification potential results indicate a total impact of around 4.64 × 10^−4^ mol H^+^ eq. kg^−1^ of concentrate. Similar to the carbon footprint, electricity consumption in NF and DF operations was the largest contributor, comprising nearly 81% of the total acidification impact. The acidification associated with electricity use primarily originates from fossil fuel combustion processes, which release sulfur dioxide (SO_2_) and nitrogen oxides (NO_x_), both significant contributors to acidification [48]. The second most significant contribution came from citric acid usage (~9%), which can release acidic substances through its production lifecycle. Wastewater treatment and membrane production had relatively low contributions, each below 5%. To reduce acidification potential effectively, transitioning to renewable and optimizing chemical use during cleaning cycles should be prioritized.

Freshwater eutrophication potential totaled approximately 5.0 × 10^−5^ kg P eq. kg^−1^ of concentrate. Unlike the previous indicators, wastewater generated during membrane cleaning emerged as the dominant contributor, accounting for around 78% of this impact. This result is due to the nutrient-rich composition of wastewater, particularly phosphorus-containing compounds, arising from cleaning agents (citric acid and NaOH), residual organic matter, and membrane washing residues. Electricity consumption, particularly during DF, represented the second major contributor (~17%), linked to emissions and nutrient runoff from electricity production processes. Other factors, such as chemical production and membrane manufacturing, had minor contributions (<5%). Reducing eutrophication impacts could be effectively addressed by improving wastewater treatment efficiency or implementing internal wastewater reuse strategies, minimizing nutrient-rich discharges to freshwater bodies.

On the other hand, water use per kilogram of concentrate was approximately 6.3 × 10^−2^ m^3^ deprived kg^−1^. NF and DF electricity demands constituted the largest share (~82%), indicating high indirect water consumption associated with electricity generation processes, especially thermoelectric power plants reliant on cooling water [49]. Direct process water, although significant in volume, exhibited a minimal relative impact due to effective internal reuse strategies implemented in the filtration and cleaning cycles. Cleaning chemicals and wastewater treatment combined contributed less than 12% of the total water use impact. Thus, reducing water use impacts would mainly involve transitioning towards renewable energy sources with lower associated water demands and further enhancing internal water reuse efficiency.

#### 3.4.1. Uncertainty Analysis

As stated above, the Monte Carlo analysis was focused on the background system, in particular on the ecoinvent datasets used, while the foreground system (pilot-scale operations) was treated deterministically due to the low variability of the data.

The results show that the climate change and acidification categories exhibited relatively low uncertainty. The average global warming potential was estimated at 0.117 kg CO_2_ eq. kg^−1^ product, with a coefficient of variation (CV) of 7.2%, suggesting high robustness of the result despite variability in background datasets such as electricity mix or chemical inputs. Similarly, the mean acidification potential was 0.000506 mol H^+^ eq. kg^−1^, with an even lower CV of 4.2%, indicating minimal influence of background variability on this category. These findings are consistent with other LCAs on food side-stream valorization, such as those by Bashiri et al. or Cadena et al. [36,50], which identified energy consumption, particularly electricity, as the dominant contributor to both GWP and acidification. These studies also reported relatively stable values for these indicators due to well-characterized background processes.

In contrast, higher variability was observed in the freshwater eutrophication indicator. The average value was 6.2 × 10^−5^ kg P eq. kg^−1^, with a CV of 22.1%. This elevated uncertainty likely stems from fluctuations in nutrient emissions linked to upstream processes, particularly the production of phosphorus-containing chemicals. A similar observation was made in the LCA by Cadena et al. [36], where nutrient emissions associated with pH-shift technology for recovering proteins from diverse fish solid side streams led to considerable uncertainty in eutrophication potential, mainly due to the variations in phosphorus emissions associated with background processes.

The water use category presented a moderate level of uncertainty, with a mean of 0.065 m^3^ deprived kg^−1^ and a CV of 11.3%. Although the uncertainty is not extreme, it reflects sensitivity to regional water scarcity factors and the balance of water withdrawals and releases modelled in the background system. Boukouvalas et al. [51] found similar patterns in meat processing valorization systems, highlighting that indirect water use from electricity generation often outweighs direct water use. This confirms the importance of energy-related upstream processes in driving the overall water consumption.

#### 3.4.2. Sensitivity Analysis

To evaluate the long-term environmental performance of the assessed mussel cooking water valorization system, a prospective sensitivity analysis was performed using the premise tool. This method integrates future background system changes consistent with the REMIND SSP2-NDC pathway for 2030, 2040, and 2050. These scenarios reflect not only a decarbonizing energy mix but also broader systemic transformations, such as increased resource efficiency, shifts in industrial technologies, and policy-driven changes across supply chains.

When comparing the prospective results with the baseline scenario, which relies on the current Spanish electricity mix and ecoinvent v3.10, a clear trend of environmental improvement is observed across multiple impact categories.

**Figure 7:**
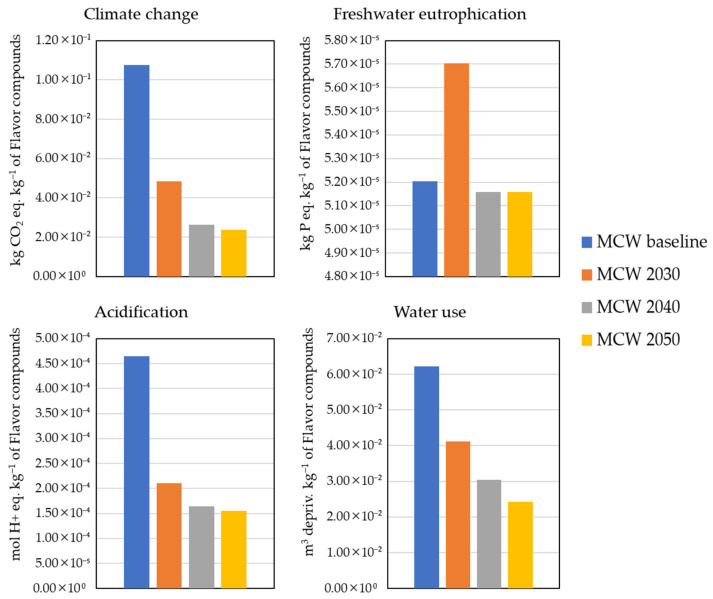
Environmental impact results for the production of 1 kg of savory compounds under the three scenarios, considering the SSP2-NDC pathway via a membrane concentration technology. MCW: mussel cooking water.

In the climate change indicator, the global warming potential decreased significantly from 0.107 kg CO_2_ eq. kg^−1^ to 0.049, 0.026, and 0.024 kg CO_2_ eq. kg^−1^ in 2030, 2040, and 2050, respectively (Figure 7). This nearly 80% reduction by 2050 reflects combined effects of lower fossil fuel reliance, improved industrial efficiencies, and cleaner electricity and heat inputs throughout the supply chain. For instance, Cadena et al. [36] reported similar reductions in GWP (up to 95%) for fish protein recovery via pH-shift methods when using future energy scenarios. The reductions reflect the combined effects of lower fossil fuel reliance, improved industrial efficiencies, and cleaner electricity and heat inputs across the value chain.

For acidification potential, the impacts dropped from 4.64 × 10^−4^ mol H^+^ eq. kg^−1^ to 2.11, 1.64 × 10^−4^, and 1.55 × 10^−4^ mol H^+^ eq. kg^−1^. This 67% reduction is likely driven by reduced emissions of precursors, such as SO_2_ and NO_x_ from electricity generation and industrial activities, as cleaner technologies are adopted.

Freshwater eutrophication impacts remained relatively stable over time, with values ranging from 5.70 × 10^−5^ to 5.16 × 10^−5^ kg P eq. kg^−1^, compared with 5.20 × 10^−5^ kg P eq. kg^−1^ in the baseline. This suggests that phosphorus emissions are less responsive to macroeconomic and energy system changes, possibly because they stem from specific chemical or agricultural processes less affected by the REMIND trajectory.

In contrast, the water use category showed strong improvements: from 6.22 × 10^−2^ m^3^ deprived kg^−1^ down to 4.12 × 10^−2^, 3.03 × 10^−2^, and 2.42 × 10^−2^ m^3^ deprived kg^−1^ in 2030, 2040, and 2050. This 61% reduction may be attributed to systemic shifts towards less water-intensive production technologies and better regional water management embedded in the scenario assumptions. Similar significant reductions in water use in future scenarios, driven by cleaner electricity production, were found by Cadena et al. [36], while Boukouvalas et al. [51] emphasized the strong link between energy systems and indirect water use in valorization contexts.

These results highlight the importance of future-oriented LCA in capturing the evolving sustainability profile of emerging technologies. The substantial improvements in most categories support the environmental viability of MCW valorization over the long term, particularly under integrated climate and policy pathways like REMIND SSP2-NDC. However, more targeted measures may be needed to address impact categories less sensitive to background transitions, such as freshwater eutrophication.

### 3.5. Limitation of the Analysis and Future Perspectives

This study provides a first environmental sustainability assessment of a novel valorization process for MCW using nanofiltration-based technology. While the results offer valuable insights into the potential environmental performance of the system, several limitations should be acknowledged.

First, the analysis is based on primary data from a single pilot-scale assay, conducted under controlled conditions. Although the pilot plant operates at a semi-industrial level, the technology readiness level (i.e., TRL 7) does not yet reflect full industrial implementation. Consequently, process efficiencies, material consumption, and energy requirements observed in the current setup may not represent those of a scaled-up or optimized system. As the technology matures, updated inventories based on larger-scale demonstrations or commercial applications should be integrated to enhance the robustness and representativeness of the LCA results. In addition, modifying membranes for pressure-driven separation opens up new possibilities for treating cooking water, since this approach minimizes membrane fouling observed when high VCF ratios are applied [52,53]. This modification can improve the efficiency and longevity of the membranes, thereby enhancing the overall sustainability of the process. Future research should explore the potential of these modifications in greater detail, evaluating their impact on both technical performance and environmental outcomes.

Second, the end-of-life treatment of nanofiltration membranes was not included in the current impact assessment. While the contribution of this element is expected to be minor compared with the overall system impacts, its inclusion in future studies could further refine the analysis, especially as more data become available regarding membrane lifespan, recycling routes, and disposal practices.

Another limitation concerns the comparative assessment of the recovered product. Due to the specific functionality of the savory-rich concentrate and the lack of directly equivalent commercial products, a meaningful benchmark or market-based comparison was not feasible. Furthermore, functional characterization of the final product is still ongoing, and therefore, a robust functional unit definition for comparative purposes could not be established. A future comparison with conventional practices, such as the current baseline of direct discharge to wastewater treatment after minimal pre-treatment, was also considered inadequate. While this alternative has lower material and energy requirements, it does not result in the production of a high-value product. Hence, it would not reflect a fair comparison from a functionality perspective.

Nevertheless, such comparative assessments are strongly recommended in future studies, once the process is technically optimized, and the end product is fully characterized, including its functional, nutritional, or sensory properties. These developments will allow the formulation of a product-oriented functional unit, enabling fair benchmarking against alternative valorization strategies or conventional additives.

While this study offers an early environmental assessment of MCW valorization via nanofiltration, its results should be interpreted within the context of an emerging technology. As the process advances toward industrialization, future LCAs should integrate updated datasets, explore sensitivity to design choices and operational conditions, and adopt a comparative framework to support decision making and potential commercialization.

## 4. Conclusions

This study had two main objectives: (i) to optimize a nanofiltration–diafiltration (NF–DF) strategy for recovering savory compounds from mussel cooking water (MCW) and (ii) to evaluate the environmental sustainability of the selected configuration using Life Cycle Assessment.

From a technical perspective, the comparative evaluation of three concentration strategies demonstrated that the selected configuration, combining a moderate NF concentration factor (VCF 10×) with an intensified DF step (VCF 20×), offers the most effective and balanced approach for industrial application. This setup achieved a high recovery of key savory compounds, particularly glutamic acid (28 mg/100 g), while maintaining high protein retention. Additionally, the generated permeates exhibited the highest COD removal efficiency (98.2%), supporting their potential reuse as cleaning liquids in mussel processing and contributing to circular economy goals. Operationally, this configuration proved to be energy-efficient and technically robust, avoiding membrane fouling and minimizing effluent volume, thus improving process stability and feasibility for scale-up.

From an environmental perspective, LCA results confirmed that electricity consumption during the NF and DF stages was the dominant contributor to most impact categories, particularly climate change, acidification, and water use. Chemical inputs (especially citric acid) also significantly contributed to acidification and eutrophication. Monte Carlo simulations indicated relatively low uncertainty for climate change and acidification categories and moderate to high uncertainty for freshwater eutrophication and water use mainly due to variability in background processes and wastewater characteristics.

The prospective sensitivity analysis using REMIND v2.1 (SSP2-NDC pathway) projected significant reductions in environmental impacts by 2050, especially for climate change (−78%) and acidification (−67%), driven by anticipated decarbonization of the energy system and improved energy efficiency. Moderate improvements were also observed for freshwater eutrophication and water use, although these categories remain sensitive to specific process emissions and regional water scarcity factors.

While the results are promising, this study is subject to limitations. The analysis was based on primary data from a semi-industrial pilot plant (TRL 7) and focused on a single optimized configuration (Assay 2). The end-of-life phase of membranes was excluded, and comparative assessments with alternative valorization routes or current disposal practices were not feasible due to lack of consistent benchmarks and full product characterization. Future research should address these gaps, expand the system boundaries, and explore the integration of foreground variability to enhance robustness as the technology progresses toward commercialization.

The selected NF–DF configuration offers a technically viable and environmentally promising strategy for the valorization of MCW. Its potential to produce high-value savory ingredients and enable water reuse supports both industrial innovation and sustainability objectives in the seafood processing sector.

## Figures and Tables

**Table 1:** Experimental assays proposed to optimize the VCF for NF and DF concentration steps.

Assay nº	Nanofiltration Step		Diafiltration Step	
	VCF	Sample	VCF	Sample
1	10	1-NF	10	1-DF
2	10	2-NF	20	2-DF
3	20	3-NF	20	3-DF

**Table 2:** Life cycle inventory associated with the production of 1 kg of savory compound concentrate (liquid concentrate 15% solids).

Inputs	Unit	Amount
Membrane module ^1^	p	1.00 × 10^0^
Process water	L	1.91 × 10^1^
Cleaning water	L	2.62 × 10^−1^
Sodium hydroxide (NaOH)	kg	7.40 × 10^−4^
Citric acid	kg	6.41 × 10^−4^
Electricity for NF ^2^	kWh	1.98 × 10^−1^
Electricity for DF ^2^	kWh	2.08 × 10^−1^
Electricity (cleaning) ^2^	kWh	1.48 × 10^−3^
Outputs		
Savory compounds	kg	1.00 × 10^0^
Wastewater (cleaning)	L	3.81 × 10^−1^
Solid waste	kg	7.84 × 10^−3^
Water recovery ^3^	L	2.74 × 10^1^

^1^ The inventory data for membrane modules are based on the NF values shown by Bordbar et al. [32] and commercial specifications. ^2^ The Spanish electricity mix was considered for this assessment. ^3^ Internal reuse.

**Table 3:** Energy consumption comparison per 1 m^3^ of MCW treatment for the 3 assays tested.

Filtration Step	Energy Consumption per m^3^ Treated (kWh m^−3^)		
	Assay 1	Assay 2	Assay 3
NF step	8.43	8.35	21.51
DF step	7.99	8.77	N.D.
Total energy per assay	16.42	17.12	21.51 ^1^

^1^ Total energy without the DF step.

**Table 4:** Process parameters for 1 m^3^ of MCW side stream treated.

Process Parameters	Assay 1	Assay 2	Assay 3
NF process flowrate (L h^−1^)	375	375	300
NF permeate volume (L)	900	900	950
DF added water (L)	900	900	950 *
DF process flowrate (L h^−1^)	500	400	350 *
DF permeate volume (L)	900	950	950 *
Final concentrate volume (L)	100	50	50 *
Total permeate volume (L)	1800	1850	1900 *
Total process time (h)	4.67	5.17	6.19 *

* Values estimated because DF assay was unfeasible due to high membrane fouling during NF step.

**Table 5:** Concentration of free amino acids (Aa) expressed in mg L^−1^ in the initial samples and resulting NF and DF concentrates.

Amino Acid	Initial Sample Aa (mg L^−1^)	Assay 1 Concentrates Aa (mg L^−1^)		Assay 2 Concentrates Aa (mg L^−1^)	
		NF 10×	DF 10×	NF 10×	DF 20×
Aspartic acid	14.3 ± 0.1	283.9 ± 4.0	173.5 ± 1.1	365.5 ± 6.7	167.3 ± 3.7
Glutamic acid	81.8 ± 1.6	567.4 ± 8.2	366.0 ± 0.9	673.7 ± 5.4	441.4 ± 9.7
Asparagine	8.0 ± 0.1	89.5 ± 5.1	41.7 ± 0.3	98.4 ± 0.9	16.0 ± 0.6
Serine	5.0 ± 0.3	70.1 ± 2.2	54.1 ± 0.5	183.7 ± 2.3	27.2 ± 0.6
Glutamine	21.6 ± 1.0	227.2 ± 5.7	62.3 ± 0.4	190.9 ± 2.3	2.2 ± 0.3
Histidine	30.0 ± 0.1	121.3 ± 3.5	66.4 ± 1.0	94.6 ± 0.1	29.7 ± 1.1
Glycine	298.7 ± 2.8	566.3 ± 10.1	106.8 ± 0.1	650.9 ± 3.4	108.9 ± 2.2
Threonine	13.3 ± 1.0	96.3 ± 1.6	57.3 ± 0.0	69.0 ± 0.2	29.6 ± 0.2
Arginine	29.6 ± 1.1	60.5 ± 22.6	37.7 ± 0.7	317.1 ± 1.3	29.9 ± 8.8
Alanine	182.2 ± 4.4	417.8 ± 8.0	100.9 ± 0.6	477.8 ± 19.3	111.2 ± 1.9
Tyrosine	26.2 ± 2.4	239.2 ± 6.9	85.5 ± 0.4	124.8 ± 0.4	92.5 ± 1.2
Cysteine	0.0 ± 0.0	0.0 ± 0.0	0.0 ± 0.0	0.0 ± 0.0	0.0 ± 0.0
Valine	5.0 ± 0.3	52.2 ± 2.4	36.6 ± 0.4	51.3 ± 0.1	60.9 ± 1.5
Methionine	28.7 ± 5.8	48.4 ± 1.8	30.7 ± 0.2	40.2 ± 5.9	34.2 ± 5.7
Norvaline	0.0 ± 0.0	0.0 ± 0.0	0.0 ± 0.0	0.0 ± 0.0	0.0 ± 0.0
Tryptophan	24.1 ± 2.0	58.2 ± 3.8	38.0 ± 0.0	67.5 ± 5.4	16.1 ± 0.0
Phenylalanine	6.5 ± 1.5	53.7 ± 4.7	38.7 ± 0.4	38.3 ± 0.4	33.0 ± 1.4
Isoleucine	5.1 ± 0.1	37.6 ± 2.8	31.4 ± 0.3	40.5 ± 0.4	42.0 ± 1.9
Leucine	12.4 ± 0.9	48.0 ± 2.8	36.0 ± 0.3	68.2 ± 0.3	75.6 ± 1.2
Lysine	56.7 ± 0.8	356.3 ± 7.8	205.5 ± 7.4	388.5 ± 3.8	106.6 ± 3.3
Hydroxyproline	276.1 ± 11.6	148.3 ± 18.2	82.0 ± 3.4	21.6 ± 4.5	77.7 ± 7.1
Sarcosine	0.0 ± 0.0	0.0 ± 0.0	0.0 ± 0.0	0.0 ± 0.0	0.0 ± 0.0
Proline	19.0 ± 9.2	34.3 ± 11.1	17.6 ± 4.3	181.7 ± 2.1	98.3 ± 8.4

Standard deviation *n* = 3.

**Table 6:** Free amino acid distribution in the resulting concentrates from Assays 1 and 2 and in the initial sample.

Amino Acid	Initial Sample Aa (%)	Assay 1 Concentrates Aa (%)		Assay 2 Concentrates Aa (%)	
		NF 10×	DF 10×	NF 10×	DF 20×
Aspartic acid	1.25 ± 0.05	7.94 ± 0.03	10.40 ± 0.13	8.76 ± 0.06	10.51 ± 0.04
Glutamic acid	7.15 ± 0.09	15.86 ± 0.07	21.94 ± 0.09	16.31 ± 0.06	27.72 ± 0.11
Asparagine	0.70 ± 0.02	2.50 ± 0.12	2.50 ± 0.00	2.38 ± 0.01	1.01 ± 0.01
Serine	0.44 ± 0.04	1.96 ± 0.04	3.24 ± 0.01	4.43 ± 0.00	1.71 ± 0.01
Glutamine	1.89 ± 0.03	6.35 ± 0.09	3.74 ± 0.00	4.60 ± 0.00	0.14 ± 0.02
Histidine	2.62 ± 0.09	3.39 ± 0.06	3.98 ± 0.03	2.31 ± 0.03	1.87 ± 0.02
Glycine	26.12 ± 0.60	15.83 ± 0.12	6.40 ± 0.05	15.81 ± 0.10	6.84 ± 0.04
Threonine	1.16 ± 0.05	2.69 ± 0.02	3.43 ± 0.02	1.68 ± 0.02	1.86 ± 0.03
Arginine	2.60 ± 0.18	1.70 ± 0.65	2.26 ± 0.05	7.71 ± 0.06	1.87 ± 0.50
Alanine	15.93 ± 0.13	11.68 ± 0.11	6.05 ± 0.00	11.19 ± 0.34	6.98 ± 0.06
Tyrosine	2.28 ± 0.14	6.69 ± 0.13	5.12 ± 0.01	3.06 ± 0.04	5.81 ± 0.08
Cysteine	0.00 ± 0.00	0.00 ± 0.00	0.00 ± 0.00	0.00 ± 0.00	0.00 ± 0.00
Valine	0.43 ± 0.01	1.46 ± 0.05	2.19 ± 0.01	1.25 ± 0.02	3.83 ± 0.01
Methionine	2.49 ± 0.43	1.35 ± 0.04	1.84 ± 0.00	1.13 ± 0.16	2.16 ± 0.41
Norvaline	0.00 ± 0.00	0.00 ± 0.00	0.00 ± 0.00	0.00 ± 0.00	0.00 ± 0.00
Tryptophan	2.11 ± 0.24	1.63 ± 0.09	2.28 ± 0.01	1.52 ± 0.11	0.52 ± 0.52
Phenylalanine	0.57 ± 0.15	1.50 ± 0.12	2.32 ± 0.04	0.92 ± 0.00	2.07 ± 0.03
Isoleucine	0.45 ± 0.01	1.05 ± 0.07	1.88 ± 0.03	0.98 ± 0.00	2.64 ± 0.05
Leucine	1.08 ± 0.04	1.34 ± 0.06	2.16 ± 0.00	1.67 ± 0.03	4.75 ± 0.05
Lysine	4.96 ± 0.09	9.96 ± 0.32	12.32 ± 0.37	9.39 ± 0.02	6.69 ± 0.04
Hydroxyproline	24.12 ± 0.24	4.15 ± 0.55	4.92 ± 0.23	0.42 ± 0.10	4.87 ± 0.32
Sarcosine	0.00 ± 0.00	0.00 ± 0.00	0.00 ± 0.00	0.00 ± 0.00	0.00 ± 0.00
Proline	1.63 ± 0.75	0.96 ± 0.30	1.05 ± 0.25	4.49 ± 0.10	6.17 ± 0.37

Standard deviation *n* = 3.

## Data Availability

Detailed data can be found in the specific publications cited.

## Abbreviations

The following abbreviations are used in this manuscript:

Aa	free amino acids
COD	Chemical Oxygen Demand
CV	coefficient of variation
DF	diafiltration
LCA	Life Cycle Assessment
MCW	mussel cooking water
MWCO	Molecular Weight Cut-Off
NF	nanofiltration
REMIND	Regional Model of Investments and Development
VCF	volumetric concentration factor
